# Open science takes on Parkinson’s disease

**DOI:** 10.7554/eLife.66546

**Published:** 2021-02-25

**Authors:** Ekemini AU Riley, Randy Schekman

**Affiliations:** 1 Aligning Science Across Parkinson’s (ASAP)Chevy ChaseUnited States; 2 Department of Molecular and Cell Biology, Howard Hughes Medical Institute, University of California, BerkeleyBerkeleyUnited States

**Keywords:** Parkinson's disease, neurodegenerative disorders, open science, philanthropy, team science, collaboration, None

## Abstract

The Aligning Science Across Parkinson’s (ASAP) initiative was set up to improve understanding of the biology underlying the onset and progression of Parkinson’s disease. With an emphasis on open science and collaboration, we have assembled a research network led by nearly 100 investigators to explore the pathology of Parkinson’s disease, and this network will soon expand to include researchers working on relevant (dys)-functional neural circuits. We have also contributed to large-scale genetics and patient cohort initiatives related to the disease. We hope that these actions, and others planned for the future, will deepen our knowledge of the molecular mechanisms underlying the origin and evolution of Parkinson’s disease and, ultimately, contribute to the development of novel therapies.

## Introduction

Parkinson’s is a debilitating disease that impacts more than six million people worldwide, and this number is expected to double by 2040 as the population ages (Dorsey et al., 2018). Patients present with myriad symptoms at varied severity and rates of worsening. Cardinal motor symptoms of tremor, slowness and stiffness often lead to a diagnosis, but can be preceded or followed by autonomic dysfunction, mood disorders, sleep problems, orthostatic hypotension and cognitive impairment. These assorted phenotypes reflect a complex biology: (a) the loss of dopaminergic neurons in the substantia nigra along with a common, but by no means consistent, observation of alpha-synuclein-dominant neuronal inclusions; (b) the identification of 90 loci associated with common risk; and (c) the dysregulation of many cellular pathways (e.g., mitochondrial function, lysosomal activity, immune activation). How Parkinson’s disease (PD) results from this myriad of signals and systems is still not well understood (Nalls et al., 2019).

**Figure 1.**
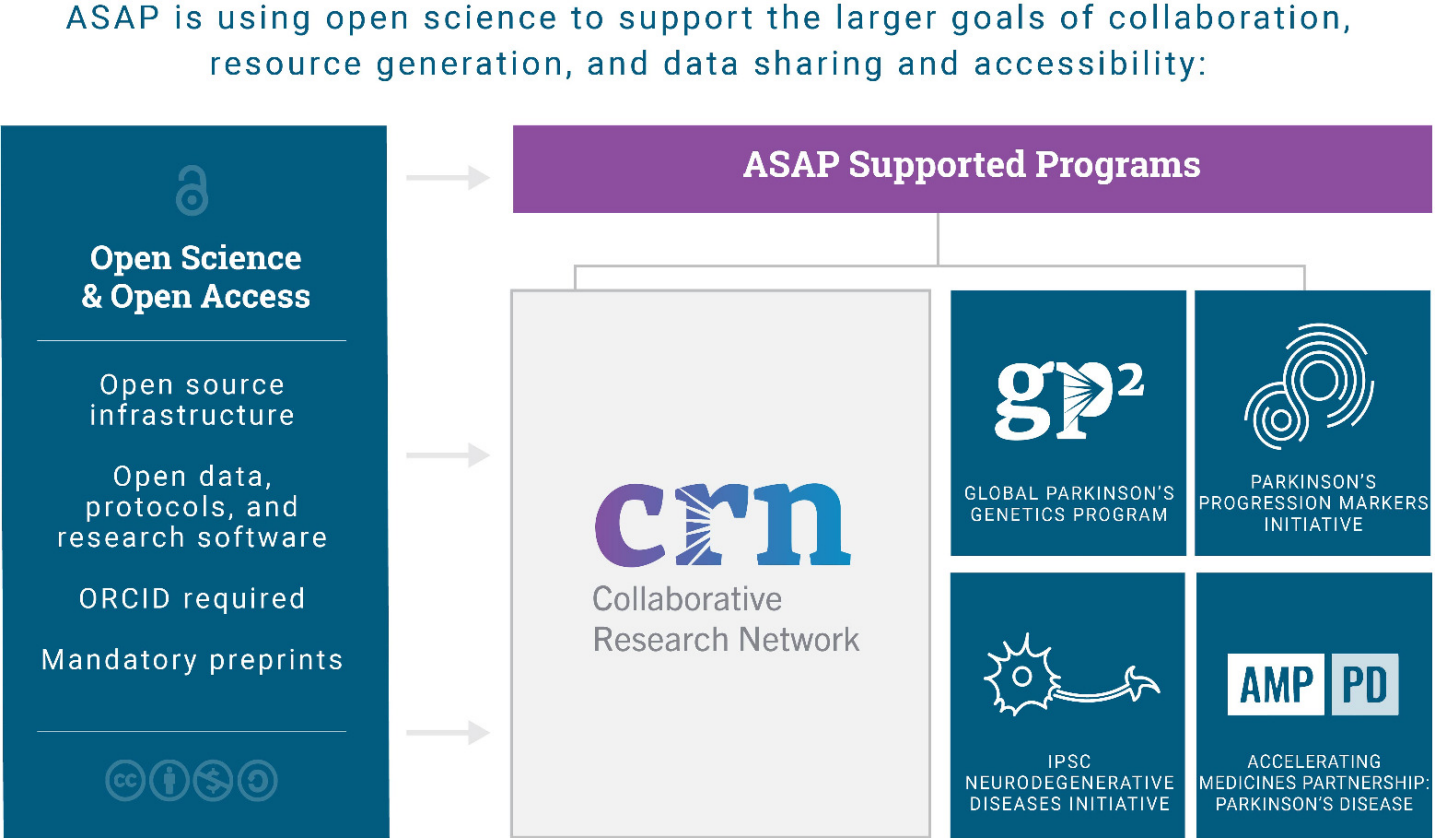
Overview of the ASAP Initiative. ASAP currently supports the Collaborative Research Network and four resource programs: the Global Parkinson’s Genetics Program (GP2); the Parkinson’s Progression Markers Initiative (PPMI); the Accelerating Medicines Partnership for Parkinson’s disease (AMP-PD) program; and the iPSC Neurodegenerative Diseases Initiative (iNDI), which is not covered in this article. Open science and open access are core tenets of ASAP's supported programs.

Planning for the Aligning Science Across Parkinson’s (ASAP) initiative began in 2017 with work to map out the funding landscape, to analyze the strategic priorities of leading public and private funders, and to identify key knowledge gaps and opportunities in PD research under the advisement of our Planning Advisory Council. Two years later we rolled out a strategic plan that was focused on four research themes and was designed to support collaboration, to generate research-enabling resources, and to democratize data (Schekman and Riley, 2019). Since then we have launched two international open calls for multidisciplinary teams to address the research themes we had identified, and launched two large-scale projects (with a third in development) to support the search for biomarker candidates and new drug targets. Working with other PD funders – notably The Michael J Fox Foundation for Parkinson’s Research (MJFF), the Parkinson’s Foundation, Parkinson’s UK, and Cure Parkinson’s Trust – has helped us to get up to speed quickly and avoid duplicative efforts. To date ASAP has committed $321m over eight years across our five active programs (see Figure 1). Together these programs will generate large amounts of data, so we have placed special emphasis on ensuring that all the data generated by ASAP-funded projects are both accessible and usable.

## Supporting collaboration and open science

In September 2020, ASAP announced the launch of its Collaborative Research Network, a team-centric network focused on large-scale basic science projects. Twenty-one teams – led collectively by 96 investigators – were selected in the first round to receive a total of $161m over three years to explore the biology of PD-associated genes (14 teams) or neuro-immune interactions (seven teams; see Box 1 for more on the science). From an initial pool of 149 self-selected teams (representing nearly 600 principal investigators), we chose finalists following two stages of review with experts from various foundations, and then a review panel of academic and industry advisers reflecting broad areas of neuroscience and immunology.

Box 1. Basic science and Parkinson’s disease.

The first 21 teams to be funded by ASAP bring established Parkinson’s disease (PD) investigators and researchers from outside of the field together to look at the genetics of the disease, and the connections between the immune system and PD. The teams are being urged to tackle thorny issues ambitiously and thoroughly, regardless of controversy, and to move past incremental gains and push the bounds of our knowledge. Sometimes this will involve investigating pathways associated with key genetic targets (such as *LRRK2*, *GBA, SNCA, PINK1* and *PRKN*, plus the many loci established from genome-wide association studies that have not yet been linked to a particular gene), and sometimes it will involve exploring lesser-known processes (such as the role of polyamine and glucosylceramide transport, the role of the microbiome in the formation of aggregates of the protein alpha synuclein, transposable elements, and the role of senescence). More information on these projects is available at http://www.parkinsonsroadmap.org/research-network.

In our second call for applications, which closed in December 2020, the focus was on exploring neuronal circuitry and brain-body connections. Specifically, we asked for applications in three areas: (a) the activity and anatomical connectivity of circuits contributing to neuronal dysfunction and degeneration; (b) cell-type-specific mechanisms and function of key neuro-modulatory effectors throughout the brain and periphery; (c) circuit dysfunction and relation to specific motor and non-motor symptoms.

**Figure 2.**
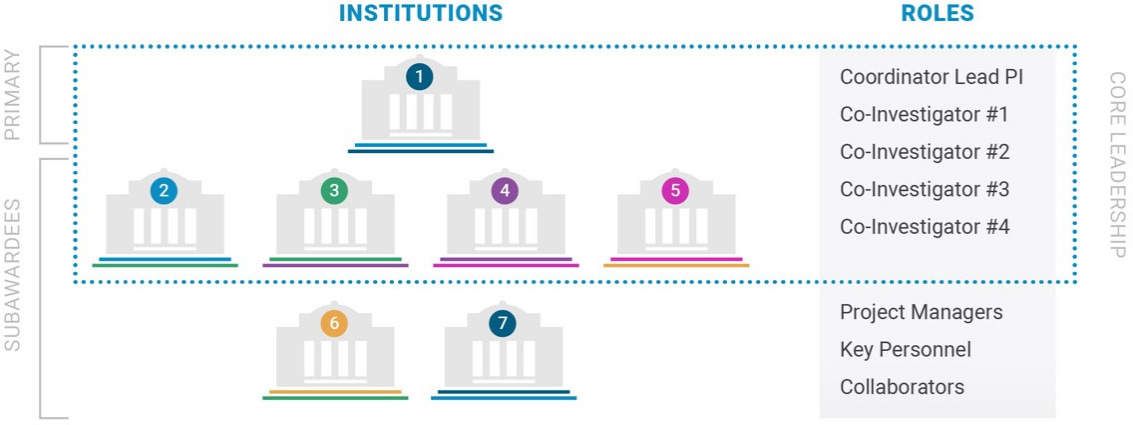
Team structure in the ASAP Collaborative Research Network. The Core Leadership of each team consists of 3–5 PIs, at least one of whom must be an early-career investigator; each team also has a Coordinator Lead PI who is responsible for overall project management and for reporting to ASAP/MJFF staff. To ensure multi-disciplinarity and geographical diversity, at least two different disciplines and between two and five institutions must also be represented across the Core Leadership.

The application guidelines for proposed teams were informed by evidence from research into 'team science' (Hall et al., 2018), and the following were used as key indicators when selecting the teams: a tendency toward collaboration (demonstrated by co-authorship, co-funded grants, or recent joint endeavors); success in previous collaborations among at least two of the proposed team leaders; and multidisciplinary team composition (see Figure 2). The 21 teams selected for funding cover a range of disciplines, geography, and experience levels: of the 96 team leaders, 35% are early-career researchers and 32% are women. Roughly half of the team leaders are based in the United States, with the other half being based in 10 different countries (including Canada, Australia and various countries in Europe). A funded full-time project manager is embedded in each team to ensure timely information exchange and close collaboration among the different labs and their dozens of graduate students, postdoctoral fellows, and research associates – currently exceeding 400 connected individuals.

ASAP is also building a virtual collaboration hub, an ongoing virtual meeting place of sorts for its grantees, which will aggregate information across the Network for ease of access, sharing, and iteration. Funded investigators will discuss ideas and queries, results, successes, and challenges to accelerate the pace of their supported studies. The virtual collaboration hub will also be used to facilitate early sharing within the Network – including results, protocols, datasets and other resources – in many cases well before publication. To encourage broader collaboration, ASAP is creating cross-team subgroups, whose resources and expertise can catalyze new experimental approaches (Tripodi et al., 2020). In addition, grantees are asked to pose open questions relating to PD research that will stimulate discussion and debate, and PhD-level facilitators with subject matter expertise, drawn from experienced scientists coordinating research through MJFF, will work across teams to identify and coordinate synergies.

ASAP has established proactive open science policies to ensure that ASAP-funded research outputs are widely available and re-usable by other research efforts. In particular, we require that manuscripts be posted on a preprint server, such as bioRxiv, before or at the point of journal submission. We also require that all datasets, code and protocols used in the research be made openly available in appropriate repositories at the time of preprint publication. Likewise, any research tools and reagents that result from ASAP-funded projects must also be publicly accessible. Our partnership with protocols.io will ensure that protocols are accessible both within the Collaborative Research Network and publicly, even in advance of publication. Moreover, in alignment with Plan S, the results of ASAP-funded research can only be published in journals that offer immediate open access with a Creative Commons CC-BY 4.0 or equivalent license. We have also joined cOAlition S – along with other international funding organizations such as the Bill and Melinda Gates Foundation, the Howard Hughes Medical Institute and Wellcome – to help grow the open-science movement.

## Generating resources

In addition to exploring the basic mechanisms responsible for the onset and progression of Parkinson's, ASAP is funding work to support the discovery and validation of new drug targets and biomarker candidates.

Led by Andrew Singleton (National Institutes of Health), the ASAP-supported Global Parkinson’s Genetics Program (GP2) has an ambitious agenda to genotype more than 150,000 volunteers in ethnically diverse populations to further understand the genetic architecture of PD. Although most of the volunteers will be from the United States and Europe, about 30% of the program's work will be in Africa, Southeast Asia and China, Central and South America, and India, as well as underrepresented populations in the United States. The inclusion of non-European ancestry data is important because the majority of disease-focused genetic studies have been performed in Europeans (Sirugo et al., 2019). Gathering deep genetic information on subjects of diverse ancestry will allow gene/variant localization through trans-ethnic fine mapping. Just as important, this diversity will provide critical information on the genetic and mechanistic differences between populations and will enable scientists and clinicians from these diverse groups to actively participate in this work.

Given the number of loci and genes identified to date, and the work that still needs to be done to understand them, one might ask why ASAP is funding work to identify more loci and genes. We believe this work provides the opportunity to understand how these disease-related proteins, genes and RNA interact in a network: if PD may be compared to a complex puzzle, it will be essential to identify and place most of these pieces in context to see the picture that emerges. Such a picture may be essential to the development of therapies that go to the core of the disease or to highlight the different paths that the disease can take, before converging on the loss of dopaminergic neurons. With $35m committed over five years, the GP2 project has begun engaging existing global consortia and cohorts to expand genetic analysis efforts with samples from those with PD, those at risk of the disease, and control volunteers. GP2 is also offering training on bioinformatics and genetic research to scientists and clinicians around the globe to enable more analyses of its library.

Some of the data feeding into GP2 comes from the Parkinson’s Progression Markers Initiative (PPMI), an international, longitudinal observational study led by Kenneth Marek (Institute for Neurodegenerative Disorders) and sponsored by MJFF. Launched in 2010 to help identify PD biomarkers, PPMI has grown to be a valuable resource for research in which biosamples (DNA, RNA, plasma, serum, whole blood, urine, saliva, and peripheral blood mononuclear cells) are paired with underlying clinical data (Marek et al., 2018; Chen-Plotkin et al., 2018). Moreover, more than 1400 volunteers – de novo PD patients, genetic and clinical risk populations, and control volunteers – are contributing clinical, imaging and biological data.

With a $125m injection of funds from ASAP, and additional contributions from its more than 30 industry partners and other donors, PPMI will expand to more than 4000 volunteers. Moreover, PPMI also plans to longitudinally assess a cohort of about 2000 unaffected individuals at high-risk of developing motor features of PD within 3–5 years due to evidence of dopaminergic deficit. This expansion will facilitate an unprecedented look at the progression of PD from the molecular scale to the clinical scale. Data from PPMI is available at ppmi-info.org, and has been downloaded more than six million times to date and users can also request access to PPMI biosamples.

ASAP has also taken a major step in support of 'data democratization' by joining the Accelerating Medicines Partnership for Parkinson’s disease (AMP-PD) program, a public-private partnership managed by the Foundation for the NIH. The program has established a portal where data is harmonized and shared from seven well-characterized PD cohorts, which includes whole-genome sequencing and clinical data on about 10,000 participants, to support the identification and validation of biomarker candidates and therapeutic targets. The portal also hosts various tools that support hypothesis-driven queries and open-ended analysis (The Lancet Neurology, 2020). Through funding support from AMP-PD, further data are being generated from these cohorts, including a targeted proteomics project and a single-nuclei RNA sequencing postmortem brain tissue project. Importantly, PPMI and GP2 are contributing data to AMP-PD.

## Looking ahead

ASAP is only just getting started with the organization of the Collaborative Research Network, and the next round of teams will be selected by Fall 2021. GP2 is gathering more cohorts, creating training tools to encourage bioinformatics competency and engagement with data, and harmonizing its data with AMP-PD, thereby making data more accessible to a wider body of investigators. PPMI has begun recruitment to identify more at-risk, de novo, and control populations to enrich the study and will also work on isogenic stem cell lines bearing PD-relevant mutations. And as the ASAP initiative continues, we will seek meaningful ways to engage patients at the basic science level and through our large-scale programs .

Parkinson’s disease, as with other neurological diseases, is a complex disorder that will require multiple approaches and many different organizations working together. Our approach is not intended to take on the field as a whole, but to strategically fill gaps where we see an opportunity to complement ongoing work. Our particular emphasis is to build strength through collaboration. By making large commitments, we hope to circumvent some of the issues that encumber traditional funding and to get philanthropy to do what it does best – that is, to shoulder risk and fill gaps – and thus help tackle a disease that has afflicted so many in the 200 years since its first clinical description.

## Data Availability

There is no primary data associated with this article.
